# Implementation of preemptive pharmacogenetic testing: progress, puzzles and priorities from an implementation science perspective

**DOI:** 10.3389/fphar.2026.1906466

**Published:** 2026-09-11

**Authors:** Rong Hu, Kaizhi Weng, Biyun Guo, Shuquan Zhuang, Junli Zhou, Zhiming Hong, Ping Lin, Chunping Wu, Zhiyuan Chen, Yue Wang, Xiaofang Wang, Huiyan Huang, Hui Zhang, Hao Zheng, Yingyi He

**Affiliations:** 1 Department of Hematology/Oncology, Fujian Children’s Hospital (Fujian Branch of Shanghai Children’s Medical Center), Fuzhou, China; 2 Department of Pharmacy, Fujian Children’s Hospital (Fujian Branch of Shanghai Children’s Medical Center), Fuzhou, China; 3 Department of Pediatric Hematology, Rheumatology and Nephrology, Zhangzhou Affiliated Hospital of Fujian Medical University, Zhangzhou, China; 4 Department of Pediatrics, The First Affiliated Hospital of Xiamen University, Xiamen, China; 5 Department of Pediatrics, Quanzhou First Hospital, Quanzhou, China; 6 Department of Cardiovascular Medicine and Hematology, Xiamen Children’s Hospital (Xiamen Branch of Children’s Hospital of Fudan University), Xiamen, China; 7 Department of Pediatrics, Quanzhou Children’s Hospital, Quanzhou, China; 8 Department of Pediatric Hematology, Fujian Institute of Hematology, Fujian Provincial Key Laboratory on Hematology, Fujian Medical University Union Hospital, Fuzhou, China; 9 Department of Pediatrics, The Chinese University of Hong Kong, Hong Kong, Hong Kong SAR, China

**Keywords:** barrier, facilitator, implementation science, multi-stakeholders, preemptive pharmacogenetics testing, PEDALs, CFIR, health equity

## Abstract

Preemptive pharmacogenetics (PGx) testing, which leverages genetic variation to predict drug response and toxicity, represents a pivotal advancement in precision medicine. By predicting drug-gene interactions and guiding precision dosing, it demonstrates significant potential to enhance drug safety and efficacy. However, its integration into routine clinical practice still faces a substantial translational gap. This review examines preemptive PGx testing implementation through an implementation science lens, moving beyond clinical utility to synthesize the implementation landscape and identify systemic integration challenges. Over the past decade, a growing body of pragmatic implementation studies has systematically cataloged barriers and facilitators across multiple levels. Adoption metrics are now relatively well-documented, however, evidence on fidelity and long-term sustainability remains scarce, and most cost-effectiveness data derive from modeling rather than implementation trials. Successful implementation of preemptive PGx testing requires synergistic strategies across multiple domains, generating robust evidence through large-scale pragmatic trials, building health informatics infrastructure, and establishing multidisciplinary service. Critical to this transformation are standardized clinical workflows, comprehensive education for healthcare providers and patients, and active participation in collaborative networks to accelerate knowledge sharing. A persistent equity gap remains that nearly all published preemptive PGx implementation programs originate from high-income countries, underscoring the urgent need for context-adapted approaches in low- and middle-income settings. Emerging implementation frameworks might offer practical guidance for embedding equity into implementation design. This paradigm shift necessitates coordinated efforts from multi-stakeholders to bridge the translational gap, ultimately enabling equitable and sustainable PGx implementation across diverse healthcare settings.

## 1 Introduction

Pharmacogenetics (PGx), a pivotal component of precision medicine, leverages individual genetic information to guide medication selection, balancing treatment efficacy and the risk of adverse drug reactions (ADR) (Dressler et al., 2018; Brown et al., 2020; Bas et al., 2024). Extensive research highlights a strong correlation between specific PGx variants and drug metabolism, therapeutic efficacy, and/or toxicity (Luzum et al., 2017; Borden et al., 2019; Varughese et al., 2020). Preemptive PGx testing, genetic testing performed before drug prescription, has gradually transitioned from concept to practice, especially with the significant reduction in genetic testing costs (Duarte et al., 2021). Large-scale randomized clinical trials (RCT) have demonstrated that preemptive PGx panel testing can significantly reduce the incidence of clinically relevant ADR, thereby affirming its potential value in clinical settings (Wang et al., 2021; Swen et al., 2023).

Despite the accumulating scientific evidence, the routine clinical integration of preemptive PGx testing remains hindered by a notable translational gap. This gap is multifaceted, reflecting uncertainties in the evidence base, the availability of actionable clinical guidelines, healthcare providers’ knowledge and attitudes, cost-effectiveness at both micro and macro levels, and patients’ willingness, acceptance, and ethical concerns (Korngiebel et al., 2017; Lim et al., 2023; Cavallari et al., 2025). Consequently, the understanding and investigation of preemptive PGx implementation are critical for propelling its bench-to-bedside translation.

Implementation science promotes the systematic uptake of evidence-based practices into routine care (Eccles and Mittman, 2006). Applying this lens, we shift the focus from whether preemptive PGx testing works to how it can be implemented in real-world settings. To this end, this review synthesizes the clinical evidence and implementation frameworks currently available, identifies the key barriers and facilitators that shape adoption, and distills practical strategies for achieving equitable and sustainable integration. Although several recent reviews have used implementation frameworks to characterize barriers and facilitators (McDermott et al., 2022), categorize delivery models (Waheed et al., 2025), or describe PGx implementation strategies in a general sense (Wu A. et al., 2024), they are often within specific countries or clinical settings. Among those focused specifically on preemptive PGx testing, most have concentrated on key evidence for clinical priorities (Chenchula et al., 2024; Valz Gris et al., 2026). This review extends the literature by offering a cross-specialty synthesis that examines implementation outcomes and strategies, foregrounds health equity, and integrates the latest evidence from implementation trials. Given that most primary studies on preemptive PGx implementation were not originally designed using implementation-science frameworks, our objective is not to produce a prospective, framework-driven implementation analysis. Rather, we apply implementation science retrospectively as an interpretive lens, a diagnostic synthesis that maps the current landscape, identifies patterns and persistent gaps, and articulates priorities for future research. We recognize that the field remains at an early transitional stage between efficacy demonstration and systematic implementation, accordingly, our contribution is directional rather than framework-proposing, intended to inform the next-generation of more deliberately theory-informed implementation studies.

## 2 Methods

For this narrative review, a targeted literature search was conducted on PubMed and Web of Science, covering publications from January 2016 to December 2025. Only English-language articles were considered. Search terms included combinations of: (“pharmacogenetic*” OR “pharmacogenomic*”) AND (“preemptive” OR “pre-emptive” OR “prospective” OR “upfront”) AND (“implement*” OR “pragmatic” OR “translation*” OR “adoption” OR “uptake” OR “barrier” OR “facilitator” OR “enabler” OR “fidelity” OR “Sustainability”). Given the narrative nature of this review, we did not apply rigid prespecified inclusion criteria for quantitative synthesis, nor did we implement dual-independent screening or PRISMA-style flow charts. Instead, article selection was guided by thematic relevance and a preference for high-quality evidence, as detailed by the following parameters. Priority was given to original research, pragmatic trials, hybrid effectiveness-implementation studies (study protocols excluded), and qualitative evaluations reporting on PGx implementation processes, barriers, facilitators, or implementation outcomes (e.g., adoption, fidelity and sustainability). Studies were included if they explicitly covered clinical implementation or translational integration of preemptive PGx testing into routine healthcare workflows. Conversely, studies were deliberately excluded if they focused solely on scientific or clinical validation of gene-drug associations, such as metabolism, efficacy, or adverse drug reactions, without engaging with the practical challenges of integrating PGx into clinical care. While such studies may employ preemptive genotyping as part of their prospective design, or recommend preemptive testing for future practice, their primary focus remains biomarker validation rather than implementation processes. These studies have been extensively reviewed elsewhere and are not the focus of this review. Foundational projects predating our search timeframe are mentioned in the narrative for historical context but were not included in the formal synthesis.

## 3 Implementation of preemptive PGx testing: evidence based practice

### 3.1 Clinical value for enhanced drug safety and precision medicine: the backbone of preemptive PGx testing

The core merit of preemptive PGx testing lies in its capacity to predict potential drug-gene interactions (DGIs) (Dressler et al., 2018; Pasternak et al., 2023), thereby enhancing the safety of pharmacotherapy. To contextualize subsequent implementation discussions, we briefly summarize clinical evidence drawn primarily from pragmatic trials and hybrid implementation studies. The best demonstration is from the PREPARE study, a preemptive PGx research conducted across seven European countries, showing a 30% reduction in clinically relevant ADR incidence when medication regimen guided by a 12-gene PGx panel (Swen et al., 2023). However, lessons from the PREPARE study highlight that implementation success can vary substantially across sites due to differences in local ethics frameworks, baseline clinical practices, and changes in case-mix over time (Guchelaar et al., 2025). For elderly patients receiving polypharmacy, a clinical pilot study also proved the feasibility of preemptive multi-gene PGx panel testing and the positive impact on prescribing patterns, healthcare resource utilization, and costs (Uber et al., 2023). Nevertheless, the small sample size and single-site design limit the generalizability of the findings. Enhanced medication therapy management (MTM) services led by clinical pharmacists, integrated with PGx testing, have also proven effective in identifying and resolving prevalent DGIs among polypharmacy patients (Reiss, 2011). Pediatric patients face distinct risks in pharmacotherapy due to their unique biophysiological development (Kearns et al., 2003) and the prevalence of off-label drug use, underscoring the critical importance of preemptive PGx application in this special population (Barker et al., 2022). An evaluation conducted at the Hospital for Sick Children in Canada showed that both targeted testing for specific drugs and preemptive testing based on whole-genome sequencing provided individualized dosing recommendations that deviated from standard regimens (Cohn et al., 2021). Furthermore, up to 80% of children in the preemptive testing cohort were recommended non-standard treatment approaches based on their genotypes (Cohn et al., 2021). However, the study did not systematically measure whether these recommendations were actually followed by clinicians, nor their downstream clinical impact. In primary care settings, retrospective analysis of large biobank data revealed that up to 60% of patients had at least one DGI (Pasternak et al., 2023), with most occurring in outpatient settings, providing strong support for promoting multi-gene panel testing. Research focusing on medically underserved populations also indicates that implementing preemptive PGx testing was not only feasible but also significantly enhanced patient satisfaction and adherence to medication therapy (Lteif et al., 2024).

Preemptive PGx testing has also proven valuable in medication selection and dosage adjustment. The function-loss alleles in *CYP2C19* impair the conversion of clopidogrel, an antiplatelet agent, to its active metabolite, consequently increasing the risk of major adverse cardiovascular events in patients undergoing percutaneous coronary intervention (Scott et al., 2013; Lee et al., 2022). A multi-center RCT proved that carriers of *CYP2C19* loss-of-function alleles had statistically higher risk of stroke at 90 days with clopidogrel than with ticagrelor (Wang et al., 2021). For 5-fluoropyrimidines and irinotecan, commonly used in gastrointestinal malignancies, as well as 6-mercaptopurine for acute lymphoblastic leukemia and inflammatory bowel disease, preemptive testing for *DPYD*/*UGT1A1* or *TPMT/NUDT15* genetic variants has become a crucial measure to define initial dose and prevent serious ADR of neutropenia and/or diarrhea (Varughese et al., 2020; White et al., 2024; Varughese et al., 2022; Amstutz et al., 2018; Relling et al., 2019). Psychiatry, particularly in the treatment of depression, has been an active area of preemptive PGx research. Given the significant inter-individual variability in antidepressant efficacy, traditional trial-and-error prescribing often leads to treatment delays and reduced patient adherence (Sperber et al., 2024). Analyzing the genotypes of key drug-metabolizing enzymes such as *CYP2D6* and *CYP2C19* could guide clinicians in selecting medications that are more likely to be effective and better tolerated (Tuteja et al., 2022; Bousman et al., 2023; Hicks et al., 2017). Nevertheless, real-world uptake of such recommendations remains low in routine clinical practice. Consequently, studies that could address the uptake challenges of preemptive PGx evidence are desperately needed.

### 3.2 Findings beyond clinical utility via preemptive PGx research

The feasibility of preemptive PGx testing was first demonstrated in the early 2010s by pioneering institutional programs such as Vanderbilt PREDICT (Pulley et al., 2012) and St. Jude PG4KDS (Hoffman et al., 2014) projects. These projects proved that preemptive PGx genotyping could be operationalized in large academic medical centers with strong institutional support, robust informatics infrastructure, and multidisciplinary collaboration. However, their resource-intensive nature also underscored the need for more generalizable and scalable implementation strategies, a challenge that later studies have continued to address. Beyond clinical utility, some hybrid-designed trials have incorporated feasibility and sustainability as research objectives. The PRIME Care study, a large-scale pragmatic RCT evaluating PGx-guided antidepressant prescribing on remission rates in major depressive disorder, concurrently explored key contextual factors influencing the dissemination of preemptive PGx testing (Vest et al., 2020; Oslin et al., 2021). The PRIME Care study demonstrated that embedding implementation science methods within a PGx RCT can generate valuable insights into provider-level barriers and facilitators, while also revealing persistent gaps between mental health and primary care providers. However, the effect on symptom remission was modest and not sustained (Oslin et al., 2022; Hain et al., 2025). A subsequent analysis suggested faster initial remission with benefit persisting over 6 months, yet the overall effect size remains modest. Providers who used PGx testing during the trial reported positive perceptions of its value, though many felt it was useful only for some patients (Vest et al., 2025). These findings suggest that while PGx testing may offer benefits for a subset of patients, particularly those at risk of DGIs, the population-level effect is limited, and the evidence base does not yet support universal implementation in psychiatric practice. Nevertheless, real-world uptake of such recommendations remains low, highlighting the need for further implementation research. A Canadian study explored the perspectives of people with lived experience and professional stakeholders on PGx testing, and the results emphasized the importance of establishing comprehensive education, technical support, and equitably accessible implementation infrastructure (Slomp et al., 2023). Notably, the qualitative design provides rich contextual insight, but the findings may not be directly transferable to other healthcare systems with different organizational structures or reimbursement models. Primary care is a critical setting for implementing preemptive PGx testing due to its broad patient reach and central role in managing chronic conditions and polypharmacy, however, PGx implementation in this environment is particularly complex given that general practitioners (GPs) typically contend with extensive knowledge demands and limited time. Qualitative studies in Australia and the UK reveal that while GPs hold positive views on PGx’s clinical utility, they commonly face barriers such as knowledge deficits, a lack of standardized guidelines, concerns of high testing costs and long turnaround time to reach PGx results (Qureshi et al., 2025; Ewasiuk et al., 2024). These findings are consistent across settings, yet both studies are limited by small sample sizes and self-selection bias, potentially overrepresenting PGx-enthusiastic clinicians.

Among all these endeavors, some breakthroughs have also been achieved. The IMPACT-GI study adopted rapid turnaround in-house testing processes and integrating PGx testing results into clinical decision support (CDS) system to overcome implementation barriers and increase preemptive testing utilization (Varughese et al., 2022). While this single-arm design demonstrated feasibility, the absence of a control group precludes direct attribution of improved utilization to the intervention itself. Nemours Children’s Health System, a large multi-state pediatric healthcare group in the U.S. serving as a model for systematic PGx implementation in pediatrics, has also developed an in-house testing platform, integrated a CDS system into the electronic health record (EHR) system, and built a comprehensive consultation clinic and multi-level education system (Cook et al., 2025). Although comprehensive, this institutional model requires substantial resources that may not be replicable in under-resourced settings. The PACIFIC-PGx clinical trial conducted in Australia investigated a pharmacist-led PGx screening service model, demonstrating high acceptance among both healthcare professionals and patients. Over 90% of healthcare providers expressed willingness to continue offering the test post-trial, affirming the acceptability and perceived sustainability (Glewis et al., 2023). Nevertheless, willingness does not directly translate to actual behavior change, future studies are still required for the sustainability assessment.

### 3.3 Application of implementation science research methodologies to enhance preemptive PGx research

Researchers have gradually adopted a spectrum of implementation science methodologies to identify, comprehend, and address the complex challenges inherent in preemptive PGx implementation. The Consolidated Framework for Implementation Research (CFIR) has been one of the most extensively utilized tools in implementation research and practice (Damschroder et al., 2009; Damschroder et al., 2022). CFIR assists in identifying barriers and facilitators across diverse contexts through five key domains, including intervention characteristics, outer setting, inner setting, characteristics of individuals, and process of implementation (Damschroder et al., 2009). CFIR has been utilized in preemptive PGx implementation studies within primary care (Qureshi et al., 2025; Ewasiuk et al., 2024), oncology (Lau-Min et al., 2022), psychiatry (Vest et al., 2020; Vest et al., 2023), and large healthcare systems (Dong et al., 2021). The Theoretical Domains Framework (TDF), developed from behavioral science, focuses on understanding healthcare providers’ (HCP) behavioral determinants (Atkins et al., 2017). TDF has been employed to develop survey instruments assessing the knowledge, attitudes, and practices of healthcare professionals and patients regarding preemptive PGx testing, thus informing targeted educational and intervention strategies (Cooper et al., 2024; Alexander et al., 2025). To translate identified barriers into actionable solutions, researchers frequently integrate CFIR or TDF with the ERIC (Expert Recommendations for Implementing Change) strategies (Waltz et al., 2014). This systematic matching process allows for the selection of the most effective interventions from a standardized repertoire of implementation strategies (Dong et al., 2021; Wiss et al., 2024). Additionally, some studies have incorporated other frameworks such as EPIS (Exploration, Preparation, Implementation, Sustainment) (Moullin et al., 2019) to guide the full life-cycle management of PGx services, from exploration to sustained operation (Varughese et al., 2022). Some other studies have leveraged the Diffusion of Innovation Theory (Dearing, 2009) to analyze factors influencing physician adoption of PGx testing (Koufaki et al., 2024). Linking PGx’s value proposition with the Triple Aim framework (Berwick et al., 2008), which encompasses improving healthcare quality, enhancing population health, and reducing *per capita* costs, further enhances the health policy implications of preemptive PGx testing to decision-makers (Sperber et al., 2024). The application of these theoretical frameworks has equipped PGx implementation research with reproducible methodologies, fostering a shift from descriptive observations toward theory-informed implementation strategies.

### 3.4 Preemptive PGx testing implementation outcomes: current evidence and gaps

Beyond clinical utility, successful implementation of preemptive PGx testing also depends on implementation outcomes of adoption, fidelity, sustainability, and cost-effectiveness, whereas, evidence for these remains uneven in current literature.

Adoption is the most frequently reported metric. In PREPARE study (Swen et al., 2023; Guchelaar et al., 2025), 69.9% of PGx-guided recommendations were accepted by clinicians, while CDS-embedded alerts achieved 64.2% adoption for *SLCO1B1*-guided simvastatin prescribing (Obeng et al., 2023). An even higher adoption rate over 80% was reported by pharmacist-led PGx practice model program (Wick et al., 2023). These results suggest that workflow-integrated, pharmacist-involved approaches have the potential to enhance preemptive PGx implementation.

By contrast, fidelity, defined as whether preemptive PGx recommendations are actually followed as intended, is largely unreported. Few studies have verified whether clinicians made correct dose adjustments or appropriate drug switches. Sustainability is also scarcely documented. As for cost-effectiveness, a systematic review (Morris et al., 2022) found that 71% of PGx studies were cost-effective or cost-saving, yet most of this evidence comes from modeling studies, not implementation trials, and data on preemptive PGx testing or multigene panels remain limited.

Overall, adoption is relatively well-documented in preemptive PGx research, however, fidelity, sustainability, and real-world cost-effectiveness data are significant gaps, reflecting both the early stage of preemptive PGx implementation research and clear directions for future work.

## 4 Implementing preemptive PGx testing: core barriers and facilitators

Unlike traditional prospective PGx research that primarily focuses on validating gene-drug associations or assessing clinical outcomes, preemptive PGx implementation studies are explicitly or co-designed to investigate the process of integrating preemptive PGx testing into routine care. A central objective of these studies is to identify and characterize the barriers and facilitators that influence adoption, uptake, and sustainability across diverse clinical settings. Table 1 summarizes original empirical implementation studies that: (1) were conducted within specific clinical specialties (or across multiple specialties), (2) explicitly reported implementation processes, barriers, facilitators, and/or implementation outcomes, (3) focused on how preemptive PGx testing is implemented in real-world clinical practice, and (4) were published between January 2016 and December 2025. We excluded studies focused solely on CDS technical implementation, system architecture, delivery-model typologies as they were separately discussed in Section 5.2. Studies that were foundational to the field but predated our search timeframe (e.g., Vanderbilt PREDICT (Pulley et al., 2012), St. Jude PG4KDS (Hoffman et al., 2014)) are referenced narratively for historical context but were not included in Table 1. The following Section 4.1, Section 4.2, Section 4.3, Section 4.4) examine these factors in greater depth across four interconnected domains guided by the CFIR framework (Damschroder et al., 2009). Specifically, we applied the first four CFIR domains of intervention characteristics, outer setting, inner setting, and characteristics of individuals to organize the barriers and facilitators identified in the literature. The fifth domain, process, was not applied because most primary studies did not report sufficient process-related data. To bridge the gap between barrier identification and intervention design, Table 2 maps the four CFIR domains to representative barriers and ERIC-informed strategy directions. This table is intended as a directional guide rather than an empirically validated matching framework. The strategy suggestions derive from the CFIR-ERIC toolbox but have not been tested against implementation outcomes in the preemptive PGx context, and their selection requires contextual adaptation to local resources, workflows, and patient populations. Accordingly, actual strategy specification must be tailored to each setting.

**TABLE 1:** Summary of key preemptive pgx implementation studies: findings, barriers, and facilitators.

No	Country	Year	Study/Program	Specialty area	Key findings	Barriers/Facilitators	Ref.
1	United States	2020	rWGS pilot in acute CVD	Cardiology	(1) Pilot study integrating rapid whole-genome sequencing in acute cardiovascular patients (2) Identified actionable PGx variants alongside pathogenic variants	Barriers: TAT, cost, interpretation complexity Facilitators: Rapid sequencing, comprehensive genomic approach	Aryan et al. (2020)
2	United States	2021	12-site early adopter survey	Cardiology	(1) Survey of 12 early-adopting institutions on clopidogrel PGx implementation (2) Consensus on CDS, pharmacist involvement, and clinical leadership support	Barriers: Variability in platforms, TAT and reimbursement Facilitators: CDS, pharmacist/expert involvement, clinical champions	Duarte et al. (2021)
3	United States	2022	Interventional cardiologist survey	Cardiology	(1) Knowledge and attitudes did not significantly influence clinicians’ willingness to follow genotype-guided recommendations	Barriers: System-level workflow challenges, individual knowledge gaps Facilitators: System-level support, automated guidance	Hoffecker et al. (2022)
4	United States	2023	SLCO1B1 CDS study	Cardiology	(1) EHR-embedded CDS alerts for SLCO1B1 genotype-guided simvastatin prescribing achieved 64.2% adoption rate	Barriers: Alert fatigue, workflow integration Facilitators: Point-of-care CDS, automated alerts	Obeng et al. (2023)
5	United States	2017	PGRN Translational Pharmacogenetics Program	Multi-specialty	(1) Cataloged pharmacogenetic implementation metrics across 8 US healthcare systems, disseminated real-world solutions to implementation barriers	Barriers: Lack of standardized data representation formats across sites, heterogeneity in CDS and genotyping platforms, inconsistent collection of cost/reimbursement and prescriber adherence data Facilitators: CPIC guidelines provided consistency across institutions, diverse CDS approaches demonstrated feasibility, Translational Pharmacogenetics Program facilitated sharing of implementation resources and best practices	Luzum et al. (2017)
6	United States	2021	VA PHASER program	Multi-specialty (VA system)	(1) Evaluated PGx implementation across initial VA test sites (2) Workforce readiness assessment	Barriers: Clinician knowledge gaps, confidence deficits Facilitators: Institutional support, CDS, educational programs	Dong et al. (2021)
7	Europe (7 countries)	2023–2025	PREPARE (U-PGx)	Multi-specialty	(1) Large European cluster-randomized crossover implementation study of 12-gene panel across seven countries (2) 30% reduction in clinically relevant ADRs in actionable patients (3) High recommendation adherence (69.9%)	Barriers: Lack of cost-effectiveness evidence and limited provider knowledge; patchy reimbursement, limited data exchange, disagreements on evidence standards, scarce accredited genotyping capacity, heterogeneous IRB assessments, varying baseline PGx practices across countries Facilitators: Educational programs, Safety Code card for point-of-care decision support, high acceptance among HCPs	Swen et al. (2023), Guchelaar et al. (2025)
8	Greece	2024	Greek physician survey	Multi-specialty	(1) Qualitative assessment of physician opinions on PGx implementation challenges and prospects	Barriers: Reimbursement, infrastructure, educational gaps, regulatory uncertainty Facilitators: Clinical utility recognition, growing evidence base	Koufaki et al. (2024)
9	Switzerland	2024	Pharmacist-led PGx services	Multi-specialty	(1) Identified strategies for overcoming barriers in pharmacist-led PGx services	Barriers: Reimbursement, infrastructure, unclear workflows, limited resources Facilitators: Pharmacist leadership, CDS, expert consultation	Wiss et al. (2024)
10	United States	2022	IMPACT-GI	Oncology (GI)	(1) Established rapid in-house testing and CDS integration to overcome implementation barriers and increase preemptive testing utilization	Barriers: Complex testing procedures, prolonged TAT, lack of standardized protocols Facilitators: Rapid in-house testing, CDS integration into EHR	Varughese et al. (2022)
11	Australia	2023	PACIFIC-PGx	Oncology	(1) Pharmacist-led PGx screening model demonstrated high acceptance (>90% providers willing to continue post-trial)	Barriers: Cost, workflow integration, lack of reimbursement Facilitators: Pharmacist-led model, multidisciplinary collaboration	Glewis et al. (2023)
12	Canada	2021	Hospital for Sick Children (SickKids) PGx Program	Pediatrics	(1) Both targeted and WGS-based preemptive testing provided individualized dosing recommendations (2) 80% of preemptive cohort recommended non-standard regimens	Barriers: Low clinician adoption of recommendations (not systematically measured in this study) Facilitators: Dual-model approach (targeted testing + preemptive WGS), tertiary pediatric setting, established PGx consultation clinic	Cohn et al. (2021)
13	Canada	2024	Canadian pediatric oncology study	Pediatrics/Oncology	(1) Identified 26 barriers and 38 facilitators across 8 centers (2) Proposed 6 evidence-based implementation strategies	Barriers: Lack of resources, clinician knowledge gaps, test TAT Facilitators: Institutional support, standardized guidelines, dedicated PGx champions	Cooper et al. (2024)
14	United States	2025	Nemours Children’s Health PGx Program	Pediatrics	(1) Developed in-house testing platform (2) CDS integrated into EHR, comprehensive consultation clinic, and multi-level education system	Barriers: Resource-intensive model Facilitators: Institutional support and funding, dedicated PGx team, long-term commitment since 2003	Cook et al. (2025)
15	United States	2024	Medically underserved population study	Primary Care	(1) Feasibility of preemptive PGx testing confirmed in medically underserved primary care setting, significant improvements in treatment satisfaction, medication adherence, and healthcare provider engagement	Barriers: Limited access, health literacy, cost Facilitators: Community engagement, participatory education	Lteif et al. (2024)
16	United States	2023	Pharmacist-led MTM with PGx	Primary Care/Pharmacy	(1) Pharmacist-led MTM integrated with PGx and CDS effectively identified and resolved prevalent DGIs in polypharmacy patients	Barriers: Lack of interdisciplinary coordination Facilitators: Pharmacist leadership, CDS integration	Wick et al. (2023)
17	Australia	2024	Australian GP qualitative study	Primary Care	(1) GPs held positive views but faced barriers including knowledge gaps, lack of guidelines, high costs, and long TAT	Barriers: Knowledge deficits, lack of standardized guidelines, high costs, long TAT Facilitators: Positive attitudes, prior PGx exposure, peer influence	Ewasiuk et al. (2024)
18	UK	2025	UK primary care mixed-methods	Primary Care	(1) Explored patient and professional perspectives (2) Identified knowledge gaps and need for cost-effectiveness evidence	Barriers: Knowledge gaps, lack of guidelines and reports, high costs Facilitators: GP willingness, patient acceptance	Qureshi et al. (2025)
19	United States	2022–2025	PRIME Care	Psychiatry	(1) Large pragmatic RCT evaluating PGx-guided antidepressant prescribing on remission rates, but modest remission benefit (2) Concurrently exploring contextual factors influencing implementation (3) Provider perceptions: positive, though testing was seen as useful only for some patients	Barriers: Limited provider knowledge and experience with PGx, uncertainty in applying results to treatment decisions, divergent views on which patients benefit most, workflow integration concerns, limited scientific evidence base Facilitators: Positive expectations of clinical value, improved patient engagement and acceptance, increased provider confidence and comfort with use over time, integration of implementation science methods	Oslin et al. (2021), Hain et al. (2025), Vest et al. (2025)
20	Canada	2023	Canadian PWLE/PHP study	Psychiatry	(1) Qualitative study exploring perspectives of people with lived experience and professional stakeholders (2) Emphasized need for education, technical support, and equitable implementation infrastructure	Barriers: Insufficient clinical utility and cost-effectiveness evidence, lack of equitable access Facilitators: Comprehensive education, technical support, accessible infrastructure	Slomp et al. (2023)

ADR, adverse drug reaction; CDS, clinical decision support; CPIC, clinical pharmacogenetics implementation consortium; CVD, cardiovascular disease; DGI, drug-gene interaction; EHR, electronic health record; GI, gastrointestinal; GP, general practitioner; HCP, healthcare professional; IMPACT-GI, implementing pharmacogenetic testing in gastrointestinal cancers; IRB, institutional review board; LMIC, low- and middle-income country; MARVEL-PIC, Minimising Adverse Drug Reactions and Verifying Economic Legitimacy - Pharmacogenomics Implementation in Children; MTM, medication therapy management; PACIFIC-PGx, Pharmacogenomic Screening for DPYD, and UGT1A1 in Cancer Patients; PGx, pharmacogenomics; PHASER, pharmacogenomic testing for veterans; PHP, professional health provider; PGRN, pharmacogenomics research network; PREPARE, preemptive pharmacogenomic testing for preventing adverse drug reactions; PRIME, care, PRecision Medicine In MEntal healthcare; PWLE, people with lived experience; RCT, randomized controlled trial; rWGS, rapid whole-genome sequencing; TAT: turnaround time; UK: united kingdom of great britain and northern ireland; U-PGx, Ubiquitous Pharmacogenomics Consortium; United States: united states of america; VA, veterans affairs; WGS, whole-genome sequencing.

**TABLE 2:** Mapping CFIR domain barriers to illustrative ERIC-informed strategy.

CFIR domain	Representative barriers	Suggested ERIC strategy directions	Implementation considerations
Intervention Characteristics	Perceived insufficient clinical utility evidence Guideline inconsistency Uncertainty about testing approach (targeted vs. panel vs. sequencing)	Conduct local consensus discussions Identify and prepare champions Develop and distribute educational materials on evidence strength	Strategy choice depends on institutional research culture, existing guideline uptake mechanisms, and local expertise
Outer and Inner Settings	Reimbursement gaps Prolonged turnaround time Limited CDS/EHR integration Lack of standardized workflows	Facilitate collection and analysis of local cost-effectiveness data Redesign workflows Develop and implement CDS tools Build interdisciplinary implementation teams	Heavily context-dependent Academic centers, community practices, and LMIC settings might require fundamentally different approaches
Characteristics of Individuals (HCPs)	Knowledge deficits Low confidence in interpretation Perceived complexity Time constraints	Conduct educational meetings and ongoing case-based training Provide clinical supervision or consultation Create point-of-care resource libraries; Integrate CDS reminders where feasible	Education alone is insufficient Workflow-embedded support (CDS, consultation, audit and feedback)
Characteristics of Individuals (Patients)	Low health literacy Privacy/confidentiality concerns Economic burden Limited understanding of PGx value	Develop plain-language patient education materials Conduct community-engaged participatory education Provide financial counseling and cost transparency Strengthen informed consent processes	Cultural tailoring and trust-building Strategies adapted to local health literacy levels and community norms

The CFIR-ERIC-informed strategy directions listed above are illustrative and derived from the CFIR-ERIC tool-box (https://cfirguide.org/guidance-tools). They are intended as potential starting points for implementation planning rather than prescriptive or empirically validated recommendations. Actual strategy selection and specification require systematic contextual assessment, stakeholder engagement, and iterative adaptation to local resources, workflows, and population needs. This table is therefore a directional guide rather than a formal CFIR-ERIC-outcomes matching framework.

CDS, clinical decision support; EHR, electronic health record; HCPs, healthcare professionals; LMIC, low- and middle-income country; PGx, pharmacogenetic.

### 4.1 Intervention characteristics: strength of clinical utility evidence and guideline standardization

While evidence in preemptive PGx testing is rapidly expanding, many clinicians still perceive a paucity of sufficient evidence regarding clinical utility, especially cost-effectiveness towards preemptive PGx testing in routine practice (Slomp et al., 2023; Lau-Min et al., 2022; Aryan et al., 2020). This evidence gap often comes from clinicians’ hesitancy in recommending preemptive PGx tests that require additional costs compared with conventional therapeutic regimens. Notably, inconsistencies among clinical guidelines issued by various professional organizations or regulatory bodies can introduce confusion into clinical practice, particularly in oncology settings (Lim et al., 2023). However, the publication of authoritative clinical practice guidelines stands as one of the most significant facilitating factors driving preemptive PGx implementation. Guidelines from the Clinical Pharmacogenomics Implementation Consortium (CPIC) and the Royal Dutch Pharmacists Association-Pharmacogenetics Working Group (DPWG) offer clear, actionable recommendations on how to tailor pharmacotherapy based on specific genotypes, serving as crucial resources for the majority of PGx implementation projects worldwide (ClinPGx, 2026; KNMP, 2026). Additionally, these guidelines provide a robust foundation for constructing CDS rules, developing educational content, and streamlining clinical workflows. Importantly, large-scale, prospective implementation studies built on established guidelines have the potential to generate the high-level evidence needed to address persistent concerns about clinical utility, and thereby accelerate the translation of preemptive PGx into practice.

Beyond guideline consistency, the choice of testing approach itself poses distinct implementation challenges. Current practice has gradually transitioned from targeted genotyping of individual genes toward multi-gene panels, with sequencing-based approaches on the horizon (Moyer and Black, 2025). Targeted genotyping (Lopes et al., 2022), while most widely adopted, captures only variants included in the test design and may miss rare but clinically relevant variants, where rare variants collectively affect a substantial number of patients. Multigene panels offer broader coverage but raise questions about which genes to include and how to manage incidental findings (Moyer and Black, 2025). Sequencing-based approaches detect the full spectrum of variants but face significant barriers in result interpretation, variant classification standardization, and clinical feasibility (McDermott et al., 2025). These technical differences translate into distinct implementation considerations, including cost, turnaround time, result complexity, and the need for specialized interpretation expertise, that must be weighed against institutional resources and clinical priorities.

### 4.2 Outer and inner settings: workflow, cost-effectiveness and insurance coverage

Structural factors at the healthcare system and organizational level present deeper challenges to preemptive PGx implementation. The absence of clear reimbursement policies and prohibitive out-of-pocket costs are universally acknowledged as primary barriers, severely restricting access to preemptive PGx test accessibility globally (Duarte et al., 2021; Glewis et al., 2023; Lau-Min et al., 2022; Wiss et al., 2024; Koufaki et al., 2024). Complex testing procedures and prolonged turnaround time undermine the utility of PGx results in time-sensitive decisions (Lau-Min et al., 2022; Hoffecker et al., 2025). Furthermore, standardized protocols for integrating PGx results into routine care pathways are often lacking (Wiss et al., 2024).​

Inadequate information technology infrastructure constitutes another bottleneck. EHR systems remain insufficiently integrated with PGx data and lack built-in CDS in many institutions, failing to provide clinicians with prompt, actionable recommendations or alerts (Wiss et al., 2024; Roosan et al., 2020). Conversely, facilitating factors include active engagement and support from high-level institutional leadership (Tuteja et al., 2022), the establishment of dedicated PGx implementation teams and/or services (Cook et al., 2025; Glewis et al., 2023), and a clear delineation of responsibilities at different workflow levels (e.g., sample collection, result interpretation, clinician-patient communication). Moreover, standardizing PGx data and enabling interoperability between systems is crucial for ensuring data sharing and long-term utility across different healthcare organizations, although this issue remains largely unaddressed in most current implementation projects (Roosan et al., 2020).

### 4.3 Characteristics of individuals (healthcare providers): knowledge, attitudes and practice behaviors

HCPs are the direct implementers of PGx clinical applications, making their knowledge, attitudes, and practice behaviors paramount to implementation success. Numerous multi-national and multi-professional survey studies consistently indicate that HCPs generally lack sufficient PGx knowledge and exhibit low confidence in their ability to interpret and apply PGx test results (Vest et al., 2020; Ewasiuk et al., 2024; Lau-Min et al., 2022; Wiss et al., 2024; Wu R. R. et al., 2024). A survey conducted within the U.S. Department of Veterans Affairs revealed that while over 70% of HCPs recognized PGx’s value in predicting drug efficacy and ADRs, only about 40% felt confident in their related knowledge and implementation skills (Wu R. R. et al., 2024). This knowledge gap directly fosters uncertainty in practice that clinicians were unsure when and/or for which patients to order testing, and modify treatment plans based on results (Vest et al., 2020; Vest et al., 2023). Despite these knowledge barriers, HCPs’ overall attitude towards PGx is largely positive, with expectations that PGx can become a powerful tool for improving patient outcomes (Ewasiuk et al., 2024; Vest et al., 2023). Facilitating factors include positive prior exposure to PGx, favorable peer influence (Ewasiuk et al., 2024), and accessible expert consultation services. Conversely, concerns regarding clinical practicality, the time cost of integrating it into daily workflows, and the perceived complexity of result interpretation represent significant practical obstacles.

### 4.4 Characteristics of individuals (patients): acceptance, information needs and ethical considerations

Patients, as the ultimate beneficiaries of preemptive PGx services, also play a crucial role in implementation success through their attitudes and needs. Research indicates that patient acceptance of PGx testing is generally favorable, though not uniformly high. A systematic review and meta-analysis found that approximately 80% of psychiatric patients express positive attitudes toward genetic testing, yet notable barriers persist, including concerns about discrimination, limited knowledge, and ethical issues (Prudhomme et al., 2026). Patients generally seek safer and more effective treatments through PGx information (Glewis et al., 2023), nonetheless, the acceptance varies across populations and contexts, and patients harbor certain concerns, primarily related to testing accuracy, the clarity of clinical benefits, privacy and confidentiality of genetic information (Lim et al., 2023), and the economic burden of testing (Sperber et al., 2024), underscoring the need for tailored education and communication strategies, transparent informed consent, financial counseling, and system-level support. Therefore, comprehensive patient education and informed consent processes are essential. Patients require an understanding of PGx testing’s value and limitations, and they should be involved in shared decision-making (Slomp et al., 2023). Studies reveal that patients desire easy-to-understand test reports and educational materials, and they tend to seek PGx information primarily from their physicians or pharmacists, again highlighting the central role of HCPs in provider-patient communication (Truong et al., 2020). Furthermore, ethical considerations, particularly in ensuring equitable access to PGx benefits for diverse racial and ethnic populations, cannot be overlooked. As current PGx data are predominantly derived from Caucasians, implementation efforts must acknowledge and address health equity issues arising from population genetic diversity (Fatumo et al., 2022; Wang et al., 2024).

Beyond equity, the ethical and governance dimensions of PGx implementation warrant careful attention. Informed consent processes must clearly communicate the scope of multi-gene panel testing, including the potential for incidental findings and the future re-use of PGx data for future prescribing decisions (Korngiebel et al., 2017). Data governance frameworks, covering data storage, access controls, secondary use, and patient preferences, remain underdeveloped across most preemptive PGx programs (Roosan et al., 2020). Furthermore, the absence of clear legal guidance on prescribing liability creates uncertainty for preemptive PGx clinical adoption (Cavallari et al., 2025). Addressing these challenges is not merely a compliance exercise but a prerequisite for building patient trust and ensuring the long-term viability of preemptive PGx services.

## 5 Integration strategies for successful implementation of preemptive PGx testing

### 5.1 Multidisciplinary team collaboration and pharmacist-led service models

Robust multidisciplinary team collaboration is essential for successful preemptive PGx testing implementation. Given the complexity of pharmacotherapy, an interdisciplinary team comprising physicians, pharmacists, genetic counselors, laboratory personnel, and information technology specialists can ensure professionalism and fluidity across all stages of PGx service delivery (Luzum et al., 2017). The role of clinical pharmacists is increasingly prominent within this collaborative model. Leveraging their expertise in clinical pharmacology, drug metabolism, and MTM, pharmacists play a central role in PGx result interpretation, providing individualized medication recommendations, and educating both clinicians and patients. Pharmacist-led or -collaborative PGx service models have been shown to be clinically feasible and highly efficient (Wu A. et al., 2024; Sperber et al., 2024; Wick et al., 2023). Integrating PGx-certified clinical pharmacists into primary care clinics has demonstrated effectiveness in identifying and resolving prevalent DGIs and optimizing medication regimens through collaborative work with physicians, specialists, and patients, with over 80% of recommendations being adopted (Wick et al., 2023). While promising, the adoption rate was based on short-term follow-up and may overstate long-term adherence. Moreover, fidelity may vary across individual pharmacists, suggesting that standardized training programs are essential to ensure consistent delivery.

Recent efforts have sought to systematically characterize how PGx testing is organized and delivered across different practice settings. A scoping review identified four predominant delivery models, including sole prescriber-led, prescriber-led within an interdisciplinary team, community pharmacist-led, and PGx consultation service (Waheed et al., 2025). These typologies highlight that the choice of delivery model shapes not only workflow design and role allocation, but also the resources and infrastructure required for successful implementation, underscoring that there is no one-size-fits-all approach.

### 5.2 Health informatics and technology infrastructure: EHR and CDS integration

A robust health informatics infrastructure is the backbone for scalable and sustainable PGx implementation. The primary task is the seamless integration of discrete, structured PGx test results into EHR system. This integration ensures that genetic information becomes a permanent component of the patients’ medical records, retrievable for any future prescribing decision. CDS is particularly crucial as it can automatically trigger point-of-care alerts when clinicians prescribe medications with potential DGIs based on the patients’ genotypes, offering concise, clear and actionable advice such as considering an alternative medication or adjusting the dose (Obeng et al., 2023; Wick et al., 2023). This proactive, workflow-embedded support could significantly enhance the utilization of PGx information and prescription adoption rates (Obeng et al., 2023). Real-world evaluations, however, indicate that alert fatigue and workflow disruption remain significant barriers to CDS effectiveness, thus requiring systematic refinement of alert logic and integration into natural prescribing workflows. Long-term CDS implementation experience from the PREDICT program at Vanderbilt over a decade has provided valuable insights into the full lifecycle of PGx-CDS, from initial design and alert logic development to system maintenance and iterative refinement (Liu et al., 2021). Similarly, the U-PGx project implemented a standardized CDS solution across more than 15 clinical sites in seven European countries, generating over 6,800 PGx reports with a median delivery time of 20 min and user satisfaction rates ranging from 63.6% to 85.2% (Blagec et al., 2022). At Mayo Clinic, CDS tools have been scaled to support 45 DGIs across more than 42,000 patients, demonstrating the feasibility of long-term, enterprise-wide CDS maintenance (Caraballo et al., 2025). These multi-institutional experiences underscore both the feasibility and the persistent challenges of CDS implementation at scale. Furthermore, developing secure, user-friendly personal health portals for patients to access and comprehend their PGx results, and to share them with local healthcare team, represents another key strategy to improve patient engagement and self-management (Truong et al., 2020). Standardizing PGx data formats and terminology to support inter-institutional information exchange and interoperability is a critical direction for future infrastructure development (Roosan et al., 2020).

### 5.3 Multi-stakeholder education and involvement

Bridging the knowledge gap for widespread PGx adoption requires a comprehensive education and training system targeted at multiple stakeholders, including healthcare providers, patients, medical and pharmacy students, and policymakers. For healthcare professionals, continuous medical education (CME), workshops, online teaching modules, and just-in-time educational resources (PGx information links integrated into CDS) can enhance their PGx knowledge and practical skills (Cook et al., 2025; Wu R. R. et al., 2024). Additionally, it is fundamental to incorporate PGx education into the core curricula of medical and pharmacy schools to cultivate the next-generation of PGx-literate healthcare professionals (Ewasiuk et al., 2024; Siamoglou et al., 2021). In contrast, patient and public education could focus on explaining basic PGx concepts, potential benefits, and limitations in comprehensible language (Slomp et al., 2023; Truong et al., 2020). Participatory PGx education programs conducted in institutions serving vulnerable populations have proven effective in elevating PGx knowledge and acceptance among community members and healthcare providers, thus promoting health equity (Johnson et al., 2020). However, education and positive attitudes do not always translate into practice. A survey of interventional cardiologists found that neither PGx knowledge nor attitudes were significantly associated with adherence to PGx-guided recommendations, suggesting that system-level factors (e.g., CDS, workflow design) may be equally critical (Hoffecker et al., 2022). This highlights the need for context-specific assessments when designing PGx implementation strategies across different specialties and settings.

### 5.4 Collaborative networks and large-scale research programs driving evidence generation and practice dissemination

Given the inherent complexity and challenges of preemptive PGx implementation, efforts from any single institution are inherently limited. Establishing and participating in collaborative networks is a critical strategy for evidence generation and knowledge sharing. Large collaborative bodies such as the Pharmacogenomics Research Network (PGRN) (Cavallari et al., 2025), the Implementing GeNomics In pracTicE Network (IGNITE) (Luzum et al., 2017; Duarte et al., 2021; Tuteja et al., 2022), and Europe’s U-PGx Alliance (van der Wouden et al., 2017) have systematically compared and evaluated the effectiveness of various implementation strategies by aggregating implementation experiences, data, and metrics from diverse healthcare systems, countries, and regions, and jointly addressing implementation challenges, providing invaluable roadmaps for subsequent practitioners. Large-scale, prospective, randomized controlled implementation research projects, either supported by these collaborative networks or independently conducted, such as PREPARE (Swen et al., 2023) and PRIME Care (Oslin et al., 2021), are essential for high-quality evidence of preemptive PGx’s clinical utility.

## 6 Current research gaps and future priorities: health equity and implementation in low- and middle-income countries (LMICs)

A striking geographic imbalance characterizes the PGx implementation literature. Almost 95% of preemptive PGx programs originated in high-income countries (HICs), with 66% in the United States alone (McDermott et al., 2022). This uneven distribution reflects a deeper reality that most LMICs are still at an early stage of PGx development, with substantial gaps in clinical evidence, technology, policy, and trained personnel (Ausi et al., 2024). Even well-established gene-drug associations from European-ancestry populations need local replication before they can inform clinical practice in LMICs. Consequently, a significant issue of health inequity arises. PGx guidelines derived from non-representative cohorts may not generalize across populations and implementation models built around sophisticated EHR systems and specialized pharmacists are often unrealistic where such infrastructure does not exist. Current CPIC and DPWG guidelines are authoritative, yet they are largely derived from data on European and East Asian populations. Allele frequencies of key pharmacogenes vary substantially across populations, yet most commercially available panels and interpretation algorithms are optimized for variants common in European-ancestry individuals. This means that patients from understudied populations may receive genotype-guided recommendations based on incomplete risk stratification. Emerging initiatives such as the Latin American Network for the Implementation and Validation of Pharmacogenomic Clinical Guidelines (RELIVAF) (Quiñones et al., 2026) seek to address this gap by producing region-specific recommendations aligned with local genetic profiles, healthcare systems, and regulatory landscapes, yet similar efforts remain scarce across most of LMICs.

The structural barriers facing LMICs extend beyond genomic evidence gaps. A recent review identified four major categories of obstacles, including scarcity of local clinical evidence, limited technology and infrastructure, gaps in policy and regulation, and shortages of human resources (Ausi et al., 2024). Cost-effectiveness data from HICs frequently cited to justify PGx implementation may not translate to LMICs due to differences in healthcare expenditure, and local cost structures. Furthermore, a scoping review found that PGx technological tools are predominantly developed in HICs, with scarce development and validation in LMIC contexts (Borbón et al., 2025). This technological dependency creates a cycle where LMICs remain consumers rather than contributors to preemptive PGx innovation, limiting both the relevance and sustainability of implemented solutions. These challenges are not confined to the HIC-LMIC divide. Even within HICs, rural and medically underserved populations face substantial barriers. A qualitative study guided by CFIR framework identified limited agency over EHR build and interoperability concerns, limited PGx familiarity across the care team and cost concerns, insufficient supporting resources and unique contextual considerations for resource-limited settings in rural and underserved U.S. healthcare settings (Bosic-Reiniger et al., 2024). These findings underscore that implementation models developed in well-resourced academic medical centers cannot be simply exported to rural or underserved community settings, even within the same country.

Workforce shortages constitute another critical barrier, particularly in LMICs where the density of trained pharmacists, genetic counselors, and clinical pharmacologists is low (Ausi et al., 2024). Informatics limitations, including the absence of standardized PGx data fields in EHRs, lack of CDS integration, and poor interoperability across systems, further impede the translation of PGx results into actionable prescribing (Bosic-Reiniger et al., 2024). Reimbursement for PGx testing remains inconsistent, with coverage varying substantially across clinical indications and payers, and cost-effectiveness evidence from HICs cannot be assumed to hold in LMIC settings with fundamentally different cost structures (Ausi et al., 2024).

If left unaddressed, the equity gaps will widen the divide in precision medicine. Looking ahead, several priorities stand out. Generating foundational evidence, replicating key PGx-drug pairs in understudied populations, is a prerequisite for meaningful implementation. Adaptation also matters. Low-cost, scalable PGx models that build on existing health platforms are more likely to succeed than wholesale importation of HICs approaches. Community-engaged approaches that involve multi-stakeholders of patients, community leaders, and frontline clinicians in the design and delivery of preemptive PGx services could enhance relevance, trust, and uptake. Task-shared workflows that leverage existing personnel, such as integrating PGx counseling into routine pharmacist consultations or using non-specialist health workers for sample collection and result communication might help mitigate workforce constraints. Capacity building, including laboratory infrastructure and workforce training, will require both international partnerships across economic regions. Economic evaluations grounded in local costs and health system contexts are needed to inform reimbursement and policy decisions. Finally, implementation research should be explicitly designed to track and address disparities in who receives and benefits from preemptive PGx testing.

To guide these efforts, emerging implementation science frameworks offer practical tools for embedding equity into research design. The recently proposed PEDALs model (Problem, Evidence-based practice, Determinants, Action, Long-term use, and Scale) provides a stepwise structure for advancing health justice in global health settings (Wang et al., 2026). Originally conceived as a teaching scaffold, the equity-adapted PEDALs model integrates fairness across all phases, ranging from problem definition and evidence appraisal, to the co-design of context-sensitive implementation strategies, and finally to the measurement of long-term outcomes using disaggregated data. For preemptive PGx implementation in LMICs, the PEDALs model offers a practical roadmap.

## 7 Conclusion

Preemptive PGx testing, as a significant advancement in precision medicine, is steadily progressing from a research frontier toward routine clinical practice, yet the translational pathway varies fundamentally by resource setting. In high-income countries, successful implementation depends on three pillars: rigorous trials to confirm clinical and economic value, robust health informatics infrastructure, and sustainable multidisciplinary service models. Yet this roadmap is not universally transferable. As highlighted by the geographic disparities, most LMICs lack even basic electronic health systems, let alone population-representative evidence generation or comprehensive clinical support infrastructures. In these settings, relying on these three pillars will not make preemptive PGx a standard tool. Instead, LMICs require foundational pillars, including building basic laboratory capacity, deploying ultra-low-cost testing strategies targeting locally prevalent priority drugs, and establishing task-shared clinical workflows. While global collaborative networks remain indispensable, context-adapted implementation must be prioritized to ensure that the promise of precision medicine does not remain confined to high-income countries but translates equitably across diverse healthcare systems worldwide. Crucially, future preemptive PGx implementation research could move toward prospective, theory-informed hybrid effectiveness-implementation designs (Type I, II, or III) that embed implementation strategies and outcome measurement from the outset. Such designs would enable the systematic mapping of CFIR-identified barriers to context-sensitive ERIC strategies and, ultimately, to quantifiable outcomes including adoption, fidelity, penetration, and sustainability, the analytical chain that the current literature cannot yet support. Achieving this will require not only methodological expertise but also institutional commitment to allocate resources for implementation process evaluation alongside clinical effectiveness assessment.
